# Short Stature is Associated with Increased Risk of Dyslipidemia in Korean Adolescents and Adults

**DOI:** 10.1038/s41598-019-50524-2

**Published:** 2019-10-01

**Authors:** Na-Kyung Oh, Yun-Mi Song, Shin-Hye Kim, Mi Jung Park

**Affiliations:** 1 Department of Pediatrics, Inje University Sanggye Paik Hospital, Seoul, Korea; 2 Department of Family Medicine, Samsung Medical Center, Sungkyunkwan University School of Medicine, Seoul, Korea

**Keywords:** Dyslipidaemias, Growth disorders

## Abstract

Adults with short stature have been previously reported to have increased risk of cardiovascular events and hyper-LDL-cholesterolemia. We aimed to assess the association between height and lipid profiles among Korean adolescents and adults. We analyzed data from the Korea National Health and Nutrition Examination Survey from 2007 to 2015, from 37,889 individuals (aged 12–59 years). In adolescents, total cholesterol (TC) and low density lipoprotein-cholesterol (LDL-C) levels had profound associations with height in both boys and girls, while high density lipoprotein-cholesterol (HDL-C) levels had an inverse association with height only in boys. Height was inversely associated with TC, triglycerides (TG), and LDL-C concentrations in men and women and positively correlated with HDL-C concentration in women. In boys, the odds ratios (ORs) for hypercholesterolemia, hypertriglyceridemia, hyper-LDL-cholesterolemia were higher for shorter subjects (ORs = 2.38~7.01), while only the OR of hyper-LDL-cholesterolemia was significantly higher in girls with short stature (OR = 3.12). In adults, the ORs for hypercholesterolemia, hypo-HDL-cholesterolemia, and hyper-LDL-cholesterolemia were significantly higher in short subjects than in tall subjects after controlling for covariates (ORs = 1.50~2.61). Also, short men showed significantly higher ORs for hypertriglyceridemia (OR = 1.85) than tall men. Short stature was significantly associated with adverse lipid profiles in both adolescents and adults.

## Introduction

Height is recognized as an important indicator that reflects genetic and environmental factors, including nutrition, hormonal regulation, pubertal development, and chronic disease during the fetal-to-adolescent period. Following the first report in 1951 of the increased risk of coronary heart disease (CHD) with short stature in adults, this inverse association between adult height and cardiovascular disease (CVD) risk has been supported by the results of several prospective large-scale studies and meta-analyses. A meta-analysis of the relationship between height and CHD revealed that short adults had ~1.5 times higher risk for CHD morbidity and mortality than adults with tall stature^1^. Another meta-analysis with 1 million subjects reported that the risk of fatal or non-fatal coronary artery disease (CAD) decreases by 8% per every 6.5 cm increase in height^2^. Similarly, previous studies from Korea have reported a negative association between height and all-cause mortality^3,4^.

Dyslipidemia is one of the major established risk factors for CVD; treatment of dyslipidemia ameliorates the risk of CVD, and dyslipidemia has been reported to be inversely associated with adult height^5–7^. Lipid levels in childhood often track to adulthood. Although CVD usually develops in later life, it is well known that the accumulation of intimal fatty streaks, which are early atherosclerotic lesions, develops from childhood in association with dyslipidemia. Furthermore, dyslipidemia often accompanies other CVD risk factors including insulin resistance and hypertension, which exacerbate atherosclerosis. The identification of risk factors for dyslipidemia in children and adolescents is therefore essential for its early detection and management and the consequent prevention of CVD in later life^8^. In Korea, the overall prevalence of dyslipidemia in adolescents aged 10–18 years is 21.7~25.2%; prevalence is reported to be two times higher (53.1~56.1%) in obese adolescents^9^. However, few studies have been conducted on the relationship between height and blood lipid concentrations in children and adolescents^10–15^. Specifically, there have been no studies on the association of dyslipidemia and height in Korean adolescents and adults. In the present study, we therefore aimed to assess the association between height and serum lipid profile in Korean adolescents and adults using data from the Korean National Health and Nutrition Examination Survey (KNHANES).

## Results

### General characteristics and lipid profiles of the study population

The mean age of the adolescents was 15.1 years, and the mean age of adult men and women were 38.9 and 39.3 years, respectively (Supplementary Table S1). Tables 1 and 2 shows the general characteristics of the study population according to the height percentile. No significant differences in age, fasting glucose, alcohol consumption, physical activity, and household income were found in adolescents by height percentile (P > 0.05). Significantly higher body mass index (BMI) (P < 0.001) in boys with higher height percentile was noted, while such association was not noted in girls. Also, significant elevation of systolic and diastolic blood pressures was found in boys (P < 0.001), while only a modest but significant elevation of diastolic pressure was found in girls (P = 0.002). No significant differences in age, BMI, blood pressures were found according to the height percentile in men (P > 0.05), while a modest but significant decreases in age, BMI, and systolic blood pressure according to the elevation of height percentile were noted in women (P < 0.05). Adults with higher height percentile showed significantly higher proportions of alcohol consumption, active physical activity, and higher household income than those with lower height percentile in both men and women (P < 0.01). The prevalence of postmenopausal women was not significantly different by height percentile (P = 0.114).

**Table 1:** General characteristics of the subjects according to the height percentile in 5,207 adolescents.

	Height percentile					P-value
	<10th	10–29th	30–69th	70–89th	≥90th	
**Boys**						
No.	267	557	1102	571	279	
Age (year)	15.1 (14.8–15.3)	15.0 (14.8–15.2)	15.2 (15.0–15.3)	15.0 (14.9–15.2)	15.1 (14.8–15.3)	0.768
Height (cm)	158.4 (157.2–159.5)	163.9 (163.2–164.5)	169.5 (169.1–169.9)	174.6 (174.1–175.1)	180.3 (179.6–181.0)	<0.001
WC (cm)	69.1 (67.6–70.5)	71.1 (70.1–72.2)	73.1 (72.3–73.9)	76.2 (75.2–77.3)	77.4 (75.9–78.9)	<0.001
BMI(kg/m^2^)	20.4 (19.9–21.0)	21.0 (20.6–21.4)	21.4 (21.1–21.7)	22.1 (21.7–22.5)	22.1 (21.6–22.7)	<0.001
Systolic BP (mmHg)	107.9 (106.0–109.8)	109.6 (108.6–110.6)	110.4 (109.6–111.2)	112.5 (111.5–113.5)	111.2 (109.8–112.6)	<0.001
Diastolic BP (mmHg)	66.2 (64.8–67.6)	67.8 (67.0–68.7)	68.5 (67.8–69.2)	69.6 (68.8–70.4)	69.2 (68.1–70.4)	<0.001
Fasting glucose (mg/dL)	90.1 (89.0–91.2)	89.5 (88.8–90.1)	89.2 (88.8–89.7)	90.2 (89.5–90.9)	89.7 (88.7–90.7)	0.184
Alcohol consumption						0.376
No	226 (79.0%)	491 (86.4%)	949 (83.2%)	497 (83.5%)	233 (82.2%)	
Occasionally	34 (19.3%)	56 (12.7%)	128 (14.8%)	64 (15.2%)	36 (14.8%)	
Excessively	3 (1.7%)	5 (0.9%)	17 (2.0%)	6 (1.3%)	6 (3.0%)	
Physical activity						0.787
No	160 (59.0%)	333 (58.9%)	634 (57.1%)	325 (57.4%)	165 (61.7%)	
Yes	102 (41.0%)	217 (41.1%)	457 (42.9%)	241 (42.6%)	109 (38.3%)	
Household income						0.399
Quartile 1	37 (14.4%)	65 (13.7%)	130 (12.9%)	59 (11.8%)	28 (12.7%)	
Quartile 2	73 (32.2%)	127 (26.5%)	241 (25.9%)	139 (26.4%)	66 (24.7%)	
Quartile 3	77 (32.2%)	185 (26.0%)	341 (25.9%)	184 (26.4%)	85 (24.7%)	
Quartile 4	80 (27.3%)	170 (28.0%)	371 (32.9%)	186 (30.0%)	96 (31.6%)	
**Girls**						
No.	236	487	976	484	248	
Age (year)	15.2 (14.9–15.5)	15.0 (14.8–15.2)	15.05 (14.9–15.2)	15.1 (14.9–15.3)	15.3 (15.0–15.6)	0.574
Height (cm)	150.0 (149.4–150.7)	155.2 (155.0–155.5)	159.9 (15938–160.1)	164.5 (164.3–164.7)	169.2 (168.9–169.5)	<0.001
WC (cm)	65.7 (64.6–66.9)	67.6 (66.7–68.5)	68.7 (69.6–71.6)	70.6 (69.6–71.6)	71.5 (70.5–72.5)	<0.001
BMI(kg/m^2^)	20.5 (20.0–21.0)	20.9 (20.6–21.4)	20.8 (20.6–21.1)	21.0 (20.6–21.4)	21.1 (20.7–21.6)	0.390
Systolic BP (mmHg)	103.1 (101.6–104.8)	103.8 (102.9–104.7)	104.3 (103.6–105.1)	105.3 (104.3–106.3)	105.1 (103.9–106.3)	0.093
Diastolic BP (mmHg)	65.8 (64.3–67.3)	65.8 (65.0–66.7)	66.3 (65.6–66.9)	67.6 (66.8–68.5)	67.9 (66.8–69.1)	0.002
Fasting glucose (mg/dL)	89.4 (86.8–92.0)	88.2 (87.4–88.9)	88.2 (87.7–88.7)	88.6 (87.9–89.3)	89.5 (88.6–90.4)	0.150
Alcohol consumption						0.481
No	212 (88.9%)	441 (90.3%)	887 (88.0%)	439 (88.9%)	219 (87.4%)	
Occasionally	17 (9.1%)	33 (7.8%)	79 (11.0%)	36 (9.4%)	24 (12.4%)	
Excessively	4 (2.0%)	5 (1.9%)	7 (1.0%)	6 (1.7%)	1 (0.2%)	
Physical activity						0.342
No	162 (70.2%)	320 (64.1%)	664 (67.2%)	329 (69.1%)	162 (62.1%)	
Yes	70 (29.8%)	158 (35.9%)	308 (32.8%)	148 (30.9%)	82 (37.9%)	
Household income						0.647
Quartile 1	33 (15.9%)	66 (15.4%)	119 (14.2%)	55 (13.4%)	25 (11.3%)	
Quartile 2	71 (32.3%)	136 (29.2%)	233 (24.6%)	109 (25.4%)	64 (28.8%)	
Quartile 3	76 (29.1%)	144 (28.7%)	305 (32.2%)	161 (32.3%)	79 (31.3%)	
Quartile 4	53 (22.7%)	135 (26.7%)	309 (29.0%)	150 (28.9%)	76 (28.6%)	

The data were present as mean (95% confidence interval) or number (%).

BMI, body mass index; BP, blood pressure; WC, waist circumference.

**Table 2:** General characteristics of the subjects according to the height percentile in 32,682 adults.

	Height percentile					P-value
	<10^th^	10–29th	30–69th	70–89th	≥90th	
**Men**						
No.	1375	2696	5463	2727	1383	
Age (yr)	39.4 (38.7–40.1)	39.0 (38.5–39.5)	38.9 (38.5–39.3)	38.6 (38.1–39.1)	38.5 (37.8–39.2)	0.306
Height (cm)	161.6 (161.4–161.8)	167.2 (167.2–167.3)	171.9 (171.9–172.0)	176.8 (176.7–176.9)	182.1 (181.9–182.3)	<0.001
Weight(kg)	62.9 (62.4–63.5)	67.6 (67.2–68.0)	71.6 (71.3–71.9)	75.8 (75.3–76.2)	81.2 (80.4–81.9)	<0.001
WC (cm)	80.9 (80.3–81.4)	82.4 (83.7–84.2)	83.9 (83.7–84.2)	85.4 (84.9–85.8)	87.4 (86.8–88.0)	<0.001
BMI(kg/m^2^)	24.0 (23.8–24.2)	24.1 (24.0–24.3)	24.2 (24.1–24.3)	24.3 (24.1–24.4)	24.5 (24.3–24.7)	0.169
Systolic BP (mmHg)	118.2 (117.3–119.1)	118.2 (117.5–118.8)	117.6 (117.2–118.1)	117.7 (117.1–118.3)	117.6 (116.7–118.4)	0.480
Diastolic BP (mmHg)	78.6 (77.9–79.3)	79.4 (78.9–79.8)	79.3 (78.9–79.6)	79.4 (78.9–79.8)	79.5 (78.9–80.1)	0.339
Fasting glucose (mg/dL)	96.7 (95.4–98.9)	96.3 (95.4–97.1)	96.9 (96.3–97.6)	96.6 (95.7–97.4)	98.3 (96.9–99.8)	0.159
Alcohol consumption						0.017
No	364 (36.5%)	590 (22.5%)	1104 (20.6%)	558 (21.1%)	272 (21.2%)	
Occasionally	701 (52.4%)	1398 (54.1%)	2955 (56.4%)	1465 (55.0%)	727 (55.3%)	
Excessively	272 (21.0%)	622 (23.4%)	1248 (23.0%)	623 (23.9%)	335 (23.4%)	
Physical activity						0.001
No	936 (69.7%)	1764 (67.9%)	3595 (67.5%)	1778 (66.9%)	844 (61.1%)	
Yes	392 (30.3%)	819 (32.1%)	1662 (32.5%)	834 (33.1%)	473 (38.9%)	
Household income						<0.001
Quartile 1	160 (12.4%)	251 (9.3%)	458 (9.1%)	149 (6.5%)	87 (6.4%)	
Quartile 2	395 (29.7%)	678 (26.9%)	1217 (23.6%)	584 (22.6%)	288 (21.2%)	
Quartile 3	424 (31.0%)	829 (31.6%)	1772 (32.5%)	910 (34.8%)	452 (33.8%)	
Quartile 4	376 (26.9%)	896 (32.1%)	1948 (34.8%)	1049 (36.2%)	544 (38.7%)	
**Women**						
No.	1932	3736	7616	3853	1901	
Age (yr)	39.5 (38.9–40.2)	39.5(39.1–40.0)	39.4 (39.1–39.8)	39.0 (18.6–39.5)	38.6 (38.0–38.2)	0.038
Height (cm)	149.6 (149.5–149.8)	154.4 (154.3–154.5)	158.9 (158.8–159.0)	163.5 (163.4–163.6)	168.4 (168.2–168.6)	<0.001
WC (cm)	75.4 (74.9–75.9)	75.7 (75.3–76.1)	76.2 (75.9–76.4)	77.2 (76.8–77.6)	77.8 (77.3–78.4)	<0.001
BMI(kg/m^2^)	23.5 (23.3–23.7)	23.1 (22.9–23.3)	22.8 (22.7–22.9)	22.7 (22.6–22.8)	22.4 (22.2–22.6)	<0.001
Systolic BP (mmHg)	111.3 (110.4–112.1)	110.2 (109.7–110.8)	110.2 (109.7–110.8)	109.7 (1109.2–110.3)	109.0 (108.3–109.8)	0.001
Diastolic BP (mmHg)	72.5 (71.9–73.1)	72.4 (71.9–72.8)	72.7 (72.4–73.0)	72.8 (72.4–73.2)	72.8 (72.3–73.3)	0.465
Fasting glucose (mg/dL)	92.7 (91.8–93.6)	93.1 (92.5–93.9)	92.9 (92.4–93.4)	92.6 (91.9–93.1)	92.6 (91.9–93.4)	0.687
Alcohol consumption						<0.001
No	1080 (55.5%)	1977 (52.3%)	3913 (50.6%)	1942 (49.3%)	979 (49.6%)	
Occasionally	728 (39.7%)	1508 (42.5%)	3149 (43.6%)	1600 (43.9%)	770 (43.1%)	
Excessively	87 (4.7%)	173 (5.2%)	422 (5.9%)	228 (6.8%)	116 (7.3%)	
Physical activity						<0.001
No	1442 (76.4%)	2697 (73.8%)	5464 (72.9%)	2652 (69.8%)	1301 (70.5%)	
Yes	443 (23.6%)	942 (26.2%)	1942 (27.1%)	1095 (30.2%)	539 (29.5%)	
Household income						<0.001
Quartile 1	253 (13.2%)	354 (9.3%)	595 (8.2%)	312 (8.7%)	136 (7.3%)	
Quartile 2	571 (30.5%)	988 (37.2%)	1884 (26.4%)	872 (23.1%)	415 (23.2%)	
Quartile 3	592 (30.5%)	1216 (27.2%)	2406 (26.4%)	1226 (23.1%)	583 (23.2%)	
Quartile 4	493 (24.8%)	1145 (30.4%)	2638 (34.0%)	1392 (35.1%)	738 (38.0%)	
Menopause						0.114
No	1426 (77.6%)	2796 (78.8%)	5770 (79.5%)	2943 (79.9%)	1496 (81.3%)	
Yes	506 (22.4%)	940 (21.2%)	1846 (20.5%)	910 (20.1%)	405 (18.7%)	

The data were present as mean (95% confidence interval) or number (%). All continuous variables were adjusted for age in women.

BMI, body mass index; BP, blood pressure; WC, waist circumference.

### Relationship between lipid profile and height

**Figure 1:**
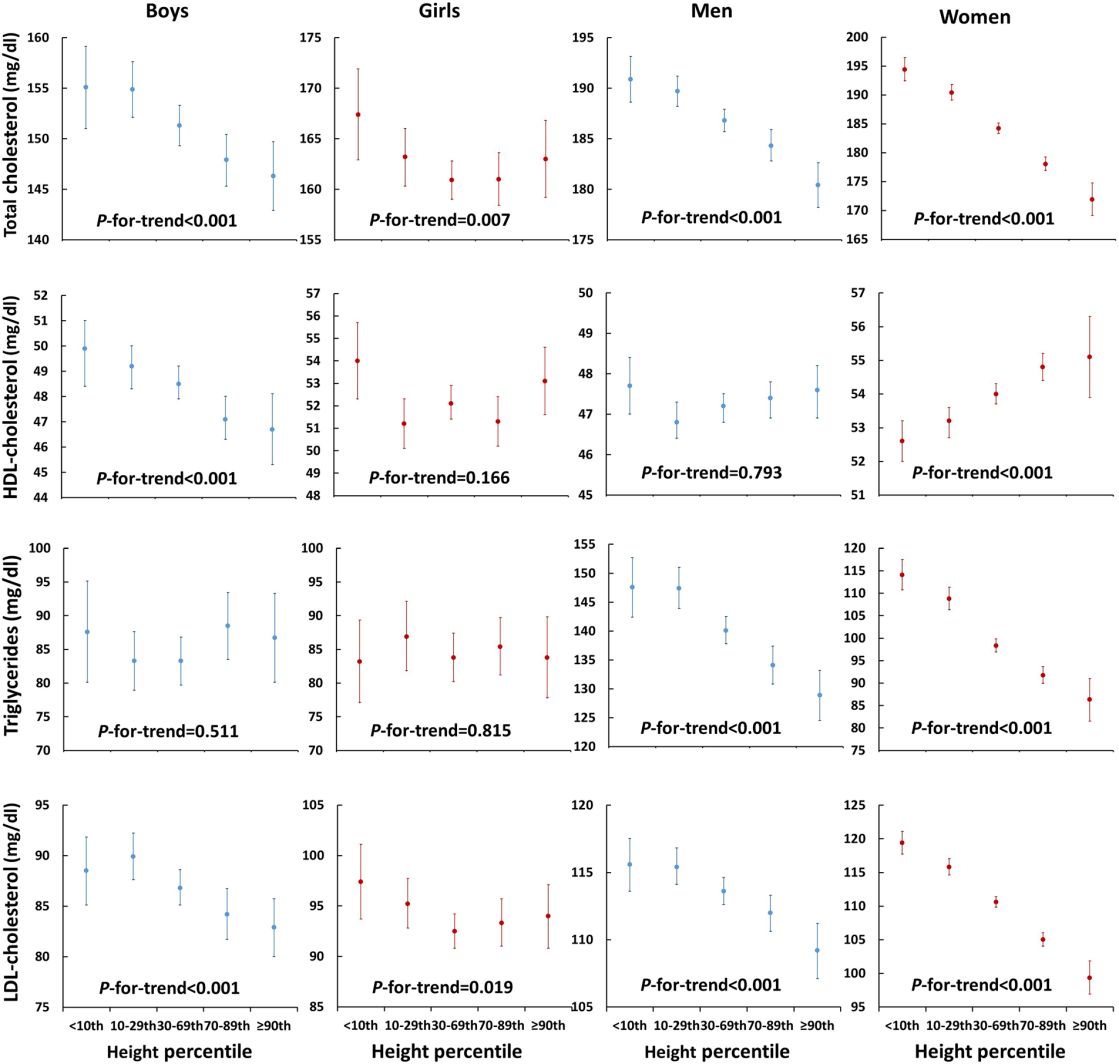
Relationship between the lipid profile and height percentile. The data were presented as mean and 95% confidence intervals (error bars).

**Figure 2:**
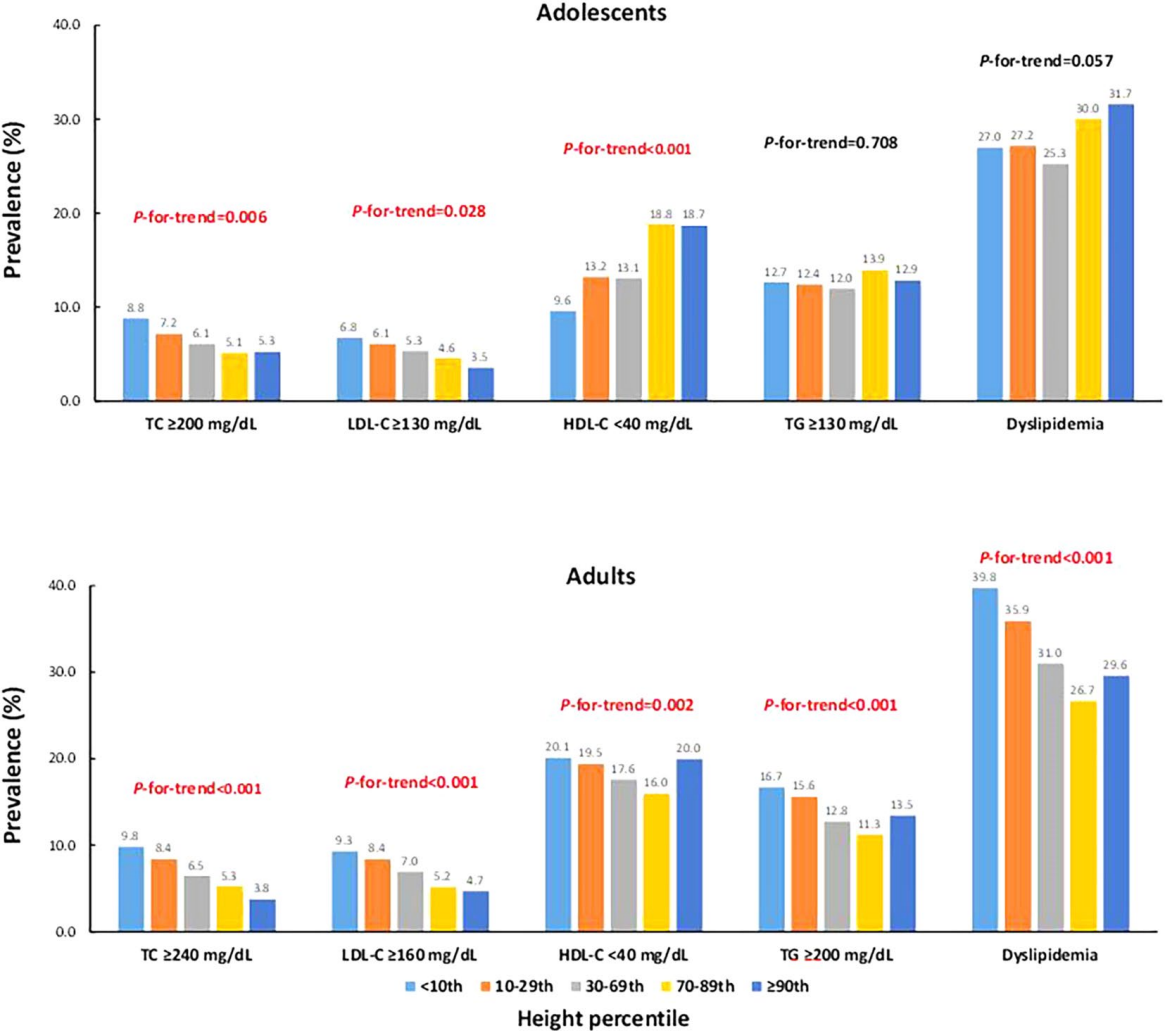
Prevalence of adverse lipid profile according to the height percentile.

Figure 1 shows the relationship between lipid profile and height percentile. In adolescents, total cholesterol (TC) and low density lipoprotein-cholesterol (LDL-C) levels showed inverse associations with height in boys and girls (P-for-trend < 0.05), while high density lipoprotein-cholesterol (HDL-C) levels had an inverse association with height only in boys (P-for-trend < 0.001). TC, triglycerides (TG), and LDL-C levels were inversely associated with height in both men and women. HDL-C levels did not significantly associate with height in men, while they positively associated in women (P-for-trend < 0.001). The prevalence of dyslipidemia according to height is shown in Fig. 2 and Supplementary Table S2. In adolescents, the prevalence of hypercholesterolemia and hyper-LDL-cholesterolemia was significantly higher in shorter subjects (P-for-trend < 0.05), while that of hypo-HDL-cholesterolemia was significantly lower in shorter subjects (P-for-trend < 0.001). No significant difference was observed in the prevalence of hypertriglyceridemia and dyslipidemia according to the height percentiles among adolescents. In adults, the prevalence of hypercholesterolemia, hyper-LDL-cholesterolemia, hypo-HDL-cholesterolemia, hypertriglyceridemia, and dyslipidemia was significantly higher in shorter subjects (P-for-trend < 0.01).

### ORs for dyslipidemia according to the height percentile

The odds ratios (ORs) and 95% confidence intervals for dyslipidemia according to the height percentile are presented in Tables 3 and 4. In boys, the ORs for hypercholesterolemia, hypertriglyceridemia, and hyper-LDL-cholesterolemia were significantly increased in shorter subjects than in taller subjects (ORs = 2.38~7.01, P-for-trend < 0.05), while the OR for hypo-HDL-cholesterolemia and dyslipidemia did not show any significant difference according to the height percentile (Table 3). In girls, only the OR for hyper-LDL-cholesterolemia was significantly increased in shorter subjects than in taller subjects (OR = 3.12, P-for-trend = 0.026). In both men and women, the ORs for hypercholesterolemia, hyper-LDL-cholesterolemia, and dyslipidemia were significantly increased in shorter subjects (ORs = 1.50~2.61, P < 0.01) than in taller subjects (height ≥90 percentile). Of note, a linear trend but an inverted J-shaped association was observed in the relationship between height and hypo-HDL-cholesterolemia in women. While the OR (95% confidence intervals) for hypo-HDL-cholesterolemia in women with heights between 10 and 29^th^ percentile was 1.54 (1.02–2.33), the OR in women with heights less than 10^th^ percentile was 1.33 (0.86–2.04). In men, the OR for hypertriglyceridemia (OR = 1.85) was significantly increased in shorter subjects than in taller subjects in men, while such an increase in OR was not statistically significant in women (Table 4).

**Table 3:** Adjusted odds ratios (95% confidence interval) for dyslipidemia in adolescents according to the height percentile.

	Height percentile					P-for-trend
	<10th	10–29th	30–69th	70–89th	≥90th	
	OR (95% CI)	OR (95% CI)	OR (95% CI)	OR (95% CI)	OR (95% CI)	
**Boys**						
TC ≥ 200 mg/dL						
Model 1	3.06 (1.01–9.24)	3.86 (1.42–10.5)	3.01 (1.13–8.01)	2.05 (0.71–5.93)	ref	0.006
Model 2	7.01 (2.26–21.7)	6.60 (2.39–18.3)	3.72 (1.38–10.1)	2.18 (0.75–6.29)	ref	<0.001
HDL-C < 40 mg/dL						
Model 1	0.42 (0.24–0.74)	0.52 (0.34–0.78)	0.58 (0.38–0.87)	0.90 (0.60–1.35)	ref	<0.001
Model 2	0.69 (0.37–1.27)	0.68 (0.43–1.06)	0.72 (0.47–1.11)	0.99 (0.63–1.54)	ref	0.271
TG ≥ 130 mg/dL						
Model 1	1.10 (0.11–0.25)	0.87 (0.62–1.96)	0.91 (0.59–1.40)	1.09 (0.67–1.78)	ref	0.997
Model 2	2.38 (1.21–4.66)	1.55 (0.87–2.12)	1.31 (0.81–2.12)	1.20 (0.68–2.08)	ref	0.048
LDL-C ≥ 130 mg/dL						
Model 1	1.56 (0.57–4.28)	2.94 (1.25–6.89)	2.17 (0.94–5.01)	1.28 (0.49–3.34)	ref	0.050
Model 2	4.35 (1.48–12.8)	5.48 (2.23–13.6)	3.05 (1.28–7.23)	1.34 (0.51–3.51)	ref	<0.001
Dyslipidemia						
Model 1	0.73 (0.46–1.14)	0.72 (0.49–1.04)	0.66 (0.46–0.94)	0.90 (0.62–1.30)	ref	0.074
Model 2	1.36 (0.81–2.27)	1.11 (0.74–1.66)	0.86 (0.59–1.26)	1.00 (0.67–1.50)	ref	0.092
**Girls**						
TC ≥ 200 mg/dL						
Model 1	1.48 (0.75–2.92)	0.88 (0.47–1.65)	0.78 (0.44–1.39)	0.75 (0.40–1.40)	ref	0.237
Model 2	1.92 (0.96–3.82)	1.07 (0.56–2.04)	0.83 (0.46–1.49)	0.80 (0.42–1.51)	ref	0.063
HDL-C < 40 mg/dL						
Model 1	0.56 (0.25–1.24)	1.05 (0.60–1.83)	0.86 (0.53–1.39)	1.27 (0.74–2.17)	ref	0.061
Model 2	0.81 (0.32–2.03)	1.30 (0.74–2.30)	1.05 (0.63–1.76)	1.35 (0.78–2.37)	ref	0.396
TG ≥ 130 mg/dL						
Model 1	1.23 (0.58–2.58)	1.40 (0.74–2.66)	1.09 (0.63–1.88)	1.13 (0.61–2.08)	ref	0.571
Model 2	1.19 (0.56–2.53)	1.42 (0.75–2.70)	1.10 (0.64–1.89)	1.15 (0.62–2.13)	ref	0.749
LDL-C ≥ 130 mg/dL						
Model 1	0.45 (0.20–1.01)	0.82 (0.39–1.72)	0.82 (0.41–1.63)	0.75 (0.37–1.53)	ref	0.225
Model 2	3.12 (1.38–7.09)	1.71 (0.81–3.64)	1.37 (0.68–2.75)	1.46 (0.70–3.04)	ref	0.026
Dyslipidemia						
Model 1	0.91 (0.57–1.45)	0.94 (0.63–1.41)	0.83 (0.58–1.20)	0.96 (0.64–1.43)	ref	0.400
Model 2	1.33 (0.80–2.21)	1.20 (0.78–1.84)	0.96 (0.65–1.43)	1.03 (0.67–1.57)	ref	0.253

Model 1: Unadjusted.

Model 2: Adjusted for age, waist circumference, systolic/diastolic blood pressure, fasting glucose levels, household income, physical activity, and alcohol consumption.

CI, confidence interval; HDL-C, high density lipoprotein-cholesterol; LDL-C, low density lipoprotein-cholesterol; OR, odd ratio; TC, total cholesterol; TG, triglycerides.

**Table 4:** Adjusted odds ratios (95% confidence interval) for dyslipidemia in adults according to the height percentile.

	Height percentile					P-for-trend
	<10th	10–29th	30–69th	70–89th	≥90th	
	OR (95% CI)	OR (95% CI)	OR (95% CI)	OR (95% CI)	OR (95% CI)	
**Men**						
TC ≥ 240 mg/dL						
Model 1	1.91 (1.32–2.74)	2.01 (1.44–2.81)	1.54 (1.13–2.12)	1.53 (1.11–2.12)	ref	<0.001
Model 2	1.84 (1.23–2.74)	1.87 (1.31–2.69)	1.51 (1.09–2.11)	1.51 (1.09–2.11)	ref	0.004
HDL-C < 40 mg/dL						
Model 1	1.27 (1.04–1.56)	1.17 (0.98–1.40)	1.14 (0.97–1.34)	1.04 (0.87–1.23)	ref	0.045
Model 2	1.50 (1.19–1.89)	1.29 (1.05–1.58)	1.26 (1.04–1.51)	1.12 (0.93–1.36)	ref	0.003
TG ≥ 200 mg/dL						
Model 1	1.58 (1.27–1.96)	1.50 (1.24–1.82)	1.27 (1.07–1.52)	1.12 (0.92–1.36)	ref	<0.001
Model 2	1.85 (1.44–2.38)	1.64 (1.32–2.05)	1.37 (1.12–1.68)	1.18 (0.94–1.47)	ref	<0.001
LDL-C ≥ 160 mg/dL						
Model 1	1.52 (1.05–2.21)	1.61 (1.17–2.21)	1.47 (1.08–1.99)	1.25 (0.90–1.73)	ref	0.001
Model 2	1.56 (1.05–2.32)	1.60 (1.14–2.27)	1.51 (1.10–2.07)	1.28 (0.92–1.78)	ref	0.026
Dyslipidemia						
Model 1	1.69 (1.41–2.02)	1.51 (1.29–1.76)	1.33 (1.15–1.54)	1.16 (0.99–1.36)	ref	<0.001
Model 2	1.93 (1.57–2.39)	1.62 (1.35–1.93)	1.44 (1.22–1.70)	1.26 (1.05–1.47)	ref	<0.001
**Women**						
LDL-C ≥ 160 mg/dL						
Model 1	6.76 (3.75–12.2)	4.75 (2.67–8.45)	3.50 (1.96–6.25)	2.22 (1.22–4.05)	ref	<0.001
Model 2	2.61 (1.41–4.81)	2.12 (1.17–3.84)	2.01 (1.11–3.63)	1.64 (0.89–3.02)	ref	<0.001
HDL-C < 40 mg/dL						
Model 1	1.48 (0.99–2.21)	1.56 (1.05–2.33)	1.22 (0.83–1.79)	0.99 (0.66–1.46)	ref	<0.001
Model 2	1.33 (0.86–2.04)	1.54 (1.02–2.33)	1.28 (0.86–1.91)	1.12 (0.74–1.68)	ref	0.003
TG ≥ 200 mg/dL						
Model 1	2.24 (1.40–3.60)	1.99 (1.26–3.13)	1.39 (0.89–2.19)	1.11 (0.69–1.77)	ref	<0.001
Model 2	1.34 (0.78–2.31)	1.35 (0.81–2.29)	1.11 (0.67–1.85)	1.06 (0.63–1.79)	ref	0.087
LDL-C ≥ 160 mg/dL						
Model 1	4.70 (2.64–8.33)	3.62 (2.06–6.35)	2.64 (1.50–4.64)	1.57 (0.87–2.83)	ref	<0.001
Model 2	1.84 (1.03–3.30)	1.71 (0.97–3.01)	1.52 (0.87–2.67)	1.19 (0.66–2.13)	ref	0.002
Dyslipidemia						
Model 1	3.09 (2.27–4.20)	2.49 (1.85–3.36)	1.80 (1.34–2.40)	1.24 (0.92–1.68)	ref	<0.001
Model 2	1.88 (1.35–2.61)	1.79 (1.31–2.45)	1.52 (1.12–2.06)	1.27 (0.93–1.73)	ref	<0.001

Model 1: Unadjusted.

Model 2: Adjusted for age, waist circumference, systolic/diastolic blood pressure, fasting glucose levels, household income, physical activity, alcohol consumption, and menopause status (for women).

CI, confidence interval; HDL-C, high density lipoprotein-cholesterol; LDL-C, low density lipoprotein-cholesterol; OR, odd ratio; TC, total cholesterol; TG, triglycerides.

## Discussion

The present study found that short stature has a significant inversely association with dyslipidemia in both Korean adolescents and adults. In adolescents, this inverse relationship between height and dyslipidemia showed sex-related differences. The risk of hypercholesterolemia, hypertriglyceridemia, and hyper-LDL-cholesterolemia was linked with height in boys, while only hyper-LDL-cholesterolemia was significantly related to height in girls. In adults, all four types of dyslipidemia were confirmed to be negatively affected by short stature in both men and women.

Since a small cross-sectional study from the 1980s reported a negative correlation between height and TC in boys^10^, a few prospective studies have analyzed the association between height gain and LDL-C levels in adolescents. The Bogalusa Heart Study reported that changes in height for five years in boys were inversely correlated with TC, LDL-C, and HDL-C levels^12^ (9). This negative correlation between the LDL-C concentration and growth rate in both boys and girls was also supported by the findings of large-scale prospective study conducted in Japan^14^. Finally, a recent study of 6,300 adolescents conducted by the West Virginia Coronary Artery Risk Detection in Appalachian Communities (CARDIAC) project reported that the risk of hyper-LDL-cholesterolemia increases significantly in the first (shortest) quartile stature compared with that in the fourth (tallest) quartile stature (OR = 1.3~3)^15^. Consistent with previous studies, the present study also found that shorter adolescents had higher TC and LDL-C levels, with significantly increased OR for hyper-LDL-cholesterolemia (OR = 3.12~4.35). Unlike those on LDL-C, studies on the association of TG with height are rare. A previous cross-sectional study in children and adolescents aged 13 to 18 years from the UK demonstrated no significant association between height and TG concentration^10^. Similarly, a longitudinal study among boys in the USA found no association between changes in TG levels and rapid height increase^12^. In contrast to this previous study, the present study found statistically higher OR for hypertriglyceridemia in shorter boys, and this association was not observed in girls. It is also important to note that obesity indices were significantly higher in boys with taller heights than in boys with shorter heights in this study population. These results suggest that the adverse effect of short stature on CVD risk in adolescents might be comparable or even stronger than obesity indices, especially for boys.

Unlike the negative correlation between height and LDL-C in both boys and girls, which is reported by most studies, reports on the relationship between height and HDL-C are inconsistent. In previous studies from the UK and USA, HDL-C levels were negatively associated with height increase in boys, but not in girls, aged 8 to 18 years^10,12^. Another study from Japan reported a negative correlation between HDL-C levels and height in both sexes in the age of 10–14 years^13^. In the present study, height was negatively correlated with HDL-C levels, only in boys, but its association with hypo-HDL-cholesterolemia was not statistically significant after adjusting for relevant covariates. In contrast with adolescents, adults with shorter height percentile showed a significantly higher risk of hypo-HDL-cholesterolemia, which might be explained by the effects of aging on HDL metabolism. Increased insulin resistance and systemic inflammation, a decline of sex hormone, and impaired lecithin cholesterol acyltransferase activity due to cellular senescence^16^ may enforce the inverse relationship between height and HDL-cholesterol in adults. The results of studies on the relationship between height and dyslipidemia/lipid concentration in adults have been inconsistent. Two prospective cohort studies from Europe reported an association between greater leg length and favorable levels of TC, LDL-C, and HDL-C at 53 years in UK adults^6^ and between taller stature and lower non-fasting TC and non-HDL-C levels in middle-aged men^5^. These two studies did not include TG concentration. In a study of individuals aged 50 years and older in China, height was negatively correlated with both LDL-C and HDL-C levels and positively correlated with TG concentration^7^. In a hospital-based study of 3,016 patients aged 30–59 years in Japan, no significant correlation was found between height and individual lipid profiles, but the OR for overall dyslipidemia in non-obese male patients was significantly decreased in taller subjects^17^. The present findings suggest that height in adulthood is favorably associated with not only LDL-C and HDL-C levels, but also TG levels. This finding is consistent with that of a recent genome-wide association study, which reported that increased height-raising allele was associated with decreased LDL-C and TG levels^18^.

An interesting fact in this study population was that taller adults tend to have higher alcohol consumption and physical activity compared with shorter adults. Higher alcohol consumption had been related to higher HDL-cholesterol in previous studies, but its beneficial effect on CVD risk is known to be alleviated by TG-elevating effect of alcohol^19^. Unlike alcohol consumption with conflicting effects on CVD risk, beneficial effects of physical activity on the lipid profiles, including lowering LDL-cholesterol and triglycerides, and increasing HDL-cholesterol have been consistently reported by previous studies^20^. To eliminate the possible favorable impact of these lifestyle factors on lipid profiles, we included these confounding factors for final adjustment. Another thing that we have to note is an inverted J-shaped association between OR for hyper-HDL-cholesterolemia and height in women. We could not find any difference in age and confounding factors between the shortest women (height <10^th^ percentile) and the next shortest women (height 10~29^th^ percentile). There has been no report of an inverted J-shaped association between height and HDL-cholesterol similar to our result. In overall, a favorable trend for hypo-HDL-cholesterolemia according to the height increase was found in women. Therefore, further research is needed to determine whether the risk of hypo-HDL-cholesterolemia is rather alleviated in severe short stature compared with mild short stature in women.

There were gender-related differences in the relationships between height and lipid profiles. In both adults and adolescents, lipid profiles in males tended to be more clearly affected by short stature. Boys with short stature showed a remarkably increased risk of both hyper-LDL-cholesterolemia (OR = 4.35) and hypertriglyceridemia (OR = 2.38), while girls with short stature showed an increased risk of hyper-LDL-cholesterolemia only (OR = 3.12). Likewise, the unfavorable effects of short stature in hypertriglyceridemia and hypo-HDL-cholesterolemia were more evident in men than in women. This gender difference can be explained by the beneficial effects of estrogen on lipid metabolism. Estrogen increases the accumulation of subcutaneous fat and restrains the increase of visceral fat, consequently allows a metabolically favorable distribution of adipose tissue^21^. Also, estrogen promotes reverse cholesterol transport process, such as hepatic secretion of cholesterol into bile, and reduces hepatic TG content through hepatic estrogen receptor α^21^.

The mechanisms supporting the link between height and dyslipidemia are not currently clear. Two hormones that play a decisive role in bone growth are growth hormone (GH) and thyroid hormone, both of which contribute to the improvement of lipid profiles. The lipolytic action of GH has been well described, particularly on visceral fat, and the administration of recombinant human GH in children and adults with GH deficiency or obesity has been reported to improve lipid metabolism by reducing TC and LDL-C levels^22,23^. In addition, it is well known that thyroid hormone plays an important role in hepatic synthesis and metabolism of fatty acids and cholesterol, and hypothyroidism leads to hypercholesterolemia and hypertriglyceridemia^24^. Therefore, it can be inferred that adolescents with adequate GH and thyroid hormone secretion are likely to have optimal growth and lipid profiles. In addition, since cholesterol is an essential component of cell membranes and steroid hormones, serum cholesterol may be lowered in individuals with a high growth velocity due to increased cholesterol consumption^12^. The skeletal system is now recognized as an important endocrine organ, which is involved in lipid metabolism. Several studies have shown that blood fatty acids are taken up by the skeletal system and are used as an energy source by the osteoblasts, thereby clearing circulating lipoproteins and non-esterified fatty acids^25^. In addition, osteocalcin, an osteoblast-derived hormone, increases the expression of the adiponectin gene in adipocytes^26^. Adiponectin causes an increase in serum HDL via inducing an increase in the hepatic production of apolipoprotein A-1 and the ATP-binding cassette transporter A1^27^. Additionally, adiponectin lowers serum TG by enhancing VLDL catabolism in skeletal muscle adipose tissue^27^. Therefore, high osteocalcin levels in adolescents with high growth rates^28^ may result in favorable lipid profiles^29^. Finally, as noted above, a recent genome-wide association study reported a correlation between genetically determined short height and increased risk of coronary artery disease, which is partly explained by an association between height-related single nucleotide polymorphisms and lipid profiles, especially those of LDL-C and TG^18^.

Our study does have some limitations. As this was a cross-sectional study, causality could not be inferred between short stature and dyslipidemia. In addition, we did not examine some confounding factors, such as pubertal stage, dietary intakes and family history of dyslipidemia. Nonetheless, to the best of our knowledge, this is the first study that reports the association of the profiles of all lipids, including TG, with height in adults as well as in adolescents, using nationally representative data. We tried to minimize the age-associated bias by using age or age-group specific height percentile. We also included various covariates including age, waist circumference, blood pressures, fasting glucose, and lifestyle factors, that are known to be potentially related to lipid profiles in the statistical analysis.

In conclusion, this study presents the evidence of short stature as associated with adverse lipid profiles in both adolescents and adults, and also suggests possible mechanisms to explain this association. Our results may help partly explain the association between short height and increased risk of CVD.

## Methods

### Study population

We analyzed data from the Korean National Health and Nutrition Examination Survey (KNHANES), conducted by the Korean Centers for Disease Control and Prevention between 2007 and 2015. This nationwide cross-sectional survey includes demographic and anthropometric information, lipid profiles and information on health-related behaviors, including alcohol intake, physical activity and household income. We limited the analyses to individuals aged 12–59 years. Among the potentially eligible 44,448 individuals, we excluded participants for whom incomplete data were available for lipid profiles and anthropometric measurements (n = 5463). Other exclusion criteria included serum triglycerides ≥400 mg/dL (n = 784); medical history of liver cirrhosis, nephrotic syndrome, or endocrine disorders; and use of medications affecting blood lipid concentrations (n = 312). Eventually, 37,889 individuals aged 12–59 years who completed blood tests for lipid profiles after an overnight fast were enrolled in this study, comprising 5,207 adolescents (2,776 boys and 2,431 girls, aged 12–18 years) and 32,682 adults (13,644 men and 19,038 women, aged 19–59 years). The KNHANES protocol was approved by an Institutional Review Board of the Korean Centers for Disease Control and Prevention (IRB No. 2007-02CON-04-P, 2008-04EXP-01-C, 2009-01CON-03-2C, 2010-02CON-21-C, 2011-02CON-06-C, 2012-01EXP-01-2C, 2013-07CON-03-4C, and 2013-12EXP-03-5C), and conducted according to the Declaration of Helsinki. All participants or their parents/guardians provided written informed consent form. This study was approved by the institutional review board of Inje University, Sanggye Paik Hospital (IRB No. SGPAIK 2019-02-005), which waived the requirement for informed consent due to the anonymity of the data obtained from the KNHANES dataset.

### Data collection and study variables

Participant height and body weight were measured to the nearest 0.1 cm using a stadiometer and 0.1 kg using a digital weight scale, respectively. BMI was calculated as the weight (kg) divided by the square of the height (m). Waist circumference was measured following a full expiration at the narrowest point between the bottom of the 10^th^ rib and the superior border of the iliac crest. Systolic and diastolic blood pressure were measured using a standard mercury sphygmomanometer on the right arm on three occasions while the participants in a sitting position after they rested for more than 10 minutes. The final blood pressure values used for analysis were the average values of three measurements.

Blood collections were performed via venipunctures from the antecubital fossa after a fasting period of at least 8 h. In 2007, serum concentrations of TC, HDL-C, TG and fasting glucose were measured enzymatically using an ADIVIA 1650 (Siemens, New York, USA). Serum LDL-C levels were calculated using Friedewald’s formula^30^. In 2008–2015, serum fasting glucose and lipid profiles, including LDL-C, were determined enzymatically using a Hitachi Automatic Analyzer 7600 (Hitachi, Tokyo, Japan). Participants were categorized as five groups according to the height percentile (<10^th^, 10–29^th^, 30–69^th^, 70–89^th^, ≥90^th^ percentile). Sex and age-specific height percentile was applied for adolescents, and sex and age group-specific (under the 30 s, 30 s, 40 s, and 50 s) height percentile was applied for adults. The sex and age-specific height percentile values were presented Supplementary Table S3. Household income was categorized into quartiles based on the equalization of income, calculated as monthly family income divided by the square root of the number of family members. Alcohol consumption was marked as “occasionally” for a subject consuming at least one drink per month in the previous year and “excessively” when the subject consumed seven drinks or more (for males) or five drinks or more (for females) on a single occasion more than 2 times a week. Alcohol consumption less than once per month in the past year was classified as “none”. Physically active persons were defined as subjects performing regular vigorous activity at least 20 minutes per session for 3 or more days per week.

### Definition of dyslipidemia

Dyslipidemia was defined as the presence of at least one of the following: hypercholesterolemia, hypertriglyceridemia, hyper-LDL-cholesterolemia, and hypo-HDL-cholesterolemia. In children and adolescents, hypercholesterolemia (≥200 mg/dL), hypertriglyceridemia (≥130 mg/dL); hyper-LDL-cholesterolemia (≥130 mg/dL), and hypo-HDL-cholesterolemia (<40 mg/dL) were defined per the National Heart, Lung, and Blood Institute’s (NHLBI) Expert Panel Guidelines^31^. In adults, hypercholesterolemia (≥240 mg/dL), hypertriglyceridemia (≥200 mg/dL), hyper-LDL-cholesterolemia (≥160 mg/dL), and hypo-HDL-cholesterolemia (<40 mg/dL) were determined according to the Korean Society of Lipid and Atherosclerosis guidelines for adults^32^.

### Statistical analysis

Statistical analyses were performed using SPSS version 21.0 (SPSS, Inc., Chicago, IL, USA). The complex samples descriptive procedure was used to evaluate the mean and standard error of scale variables. The complex samples crosstabs procedure was used for categorical or ordinal variables. The complex samples general linear model was used to compare serum concentrations of lipid profiles according to the height percentile and to calculate P values for trend. Multiple logistic regression analyses were used to calculate the ORs for dyslipidemia according to the height percentile after adjusting for covariates including age, waist circumference, systolic/diastolic blood pressure, fasting glucose, household income (quartile 1, 2, 3, 4), physical activity (yes, no), alcohol consumption (no, occasionally, excessively), and menopausal status (yes, no). TG levels were log-transformed owing to their skewed distribution. For all analyses, P-values were two-tailed and P < 0.05 was considered statistically significant.

## Supplementary information

Supplementary Information
