# Supplementary material for: Short Stature is Associated with Increased Risk of Dyslipidemia in Korean Adolescents and Adults
Source: Sci Rep. 2019 Oct 1;9:14090. doi: 10.1038/s41598-019-50524-2 (PMC6773853; doi:10.1038/s41598-019-50524-2)
Supplement: Supplementary file 1 — Supplementary Information [file 41598_2019_50524_MOESM1_ESM.pdf]

Supplementary Information

Short Stature is Associated with Increased Risk of Dyslipidemia in Korean Adolescents and Adults

Na-Kyung Oh[1], Yun-Mi Song[2], Shin-Hye Kim[1,*], Mi Jung Park[1,*]

[1]Department of Pediatrics, Inje University Sanggye Paik Hospital, Seoul, Korea.

[2]Department of Family Medicine, Samsung Medical Center, Sungkyunkwan University School of Medicine, Seoul, Korea.

Supplementary Table S1. General characteristics of the subjects

	Adolescents (N=5207)			Adults (N=32682)		
	Boys (N=2776)	Girls (N=2431)	P-value	Men (N=13644)	Women (N=19038)	P-value
**Age**	15.1±0.1	15.1±0.1	0.931	38.9±0.1	39.3±0.1	0.002
**Height (cm)**	169.3±0.2	159.8±0.1	<0.001	172.0±0.1	158.9±0.1	<0.001
**WC (cm)**	73.4±0.2	68.8±0.2	<0.001	84.0±0.1	76.4±0.1	<0.001
**BMI (kg/m^2)**	21.5±0.1	20.9±0.1	<0.001	24.2±0.1	22.9±0.1	<0.001
**Alcohol consumption**			<0.001			<0.001
No	2396 (83.4%)	2198 (88.7%)		2888 (21.7%)	9891 (51.1%)	
Occasionally	318 (14.9%)	189 (10.0%)		7246 (55.2%)	7755 (43.0%)	
Excessively	37 (1.7%)	23 (1.3%)		3100 (23.1%)	1026 (6.0%)	
**Physical activity**			<0.001			<0.001
No	1617 (58.2%)	1637 (66.8%)		8917 (67.0%)	13556 (72.6%)	
Yes	1126 (41.8%)	766 (33.2%)		4180 (33.0%)	4961 (27.4%)	
**Household income**			0.263			0.006
Quartile 1	319 (13.0%)	298 (14.2%)		1105 (8.7%)	1650 (9.0%)	
Quartile 2	646 (26.5%)	613 (26.9%)		3162 (24.4%)	4730 (26.0%)	
Quartile 3	872 (29.9%)	765 (31.1%)		4387 (32.8%)	6023 (32.1%)	
Quartile 4	903 (30.6%)	723 (27.9%)		4813 (34.2%)	6406 (33.0%)	

The data were present as mean±SE or number (%).

BMI, body mass index; WC, waist circumference

Supplementary Table S2. Prevalence of adverse lipid profile according to the height percentile

	Height percentile					
	**<10th**	**10-29th**	**30-69th**	**70-89th**	**≥90th**	**P-for-trend**
Adolescents (N=5,207)						
No.	503	1044	2078	1055	527	
TC ≥200 mg/dL	43 (8.8%)	78 (7.2%)	123 (6.1%)	61 (5.1%)	27 (5.3%)	0.006
LDL-C ≥130 mg/dL	33 (6.8%)	64 (6.1%)	110 (5.3%)	52 (4.6%)	21 (3.5%)	0.028
HDL-C <40 mg/dL	49 (9.6%)	144 (13.2%)	286 (13.1%)	184 (18.8%)	96 (18.7%)	<0.001
TG ≥130 mg/dL	60 (12.7%)	130 (12.4%)	251 (12.0%)	137 (13.9%)	65 (12.9%)	0.708
Dyslipidemia	130 (27.0%)	289 (27.2%)	537 (25.3%)	310 (30.0%)	161 (31.7%)	0.057
Adults (N=32,682)						
No.	3307	6432	13079	6580	3284	
TC ≥240 mg/dL	369 (9.8%)	569 (8.4%)	911 (6.5%)	352 (5.3%)	136 (3.8%)	<0.001
LDL-C ≥160 mg/dL	349 (9.3%)	583 (8.4%)	649 (7.0%)	346 (5.2%)	168 (4.7%)	<0.001
HDL-C <40 mg/dL	650 (20.1%)	1216 (19.5%)	2257 (17.6%)	1060 (16.0%)	663 (20.0%)	0.002
TG ≥200 mg/dL	534 (16.7%)	938 (15.6%)	1599 (12.8%)	711 (11.3%)	438 (13.5%)	<0.001
Dyslipidemia	1346 (39.8%)	2318 (35.9%)	4062 (31.0%)	1757 (26.7%)	983 (29.6%)	<0.001

The Data were presented in number (%).

HDL-C, high density lipoprotein-cholesterol; LDL-C, low density lipoprotein-cholesterol; TC, total cholesterol; TG, triglycerides

Supplementary Table S3. Sex and age-specific mean and specified percentile values (cm) of height in participants

	Mean	Height percentile			
		10 percentile	30 percentile	70 percentile	90 percentile
**Adolescents**					
**Boys**					
12 yr	157.1	145.8	152.1	161.5	167.4
13 yr	163.5	153.7	161.0	167.8	172.8
14 yr	168.9	161.2	166.1	172.1	176.6
15 yr	172.2	165.4	169.3	174.9	179.0
16 yr	173.1	166.3	169.9	176.2	181.4
17 yr	174.0	166.9	170.8	177.4	182.2
18 yr	174.2	167.8	171.1	177.0	181.7
**Girls**					
12 yr	155.7	147.5	153.2	158.9	163.5
13 yr	158.2	151.9	155.6	161.1	165.5
14 yr	159.8	153.5	157.1	162.3	167.0
15 yr	160.9	154.2	158.3	163.8	167.4
16 yr	160.8	154.8	157.9	163.4	167.5
17 yr	161.6	154.6	158.4	164.5	168.3
18 yr	160.9	153.2	158.7	164.5	168.2
**Adults**					
**Male**					
<30s	174.5	167.2	171.6	177.6	181.8
30s	173.4	166.0	170.4	176.3	180.9
40s	171.0	163.9	168.0	173.7	178.4
50s	168.5	161.2	165.6	171.6	175.7
**Female**					
<30s	161.5	154.4	158.7	164.3	168.5
30s	160.1	153.4	157.4	162.9	167.0
40s	158.2	151.5	155.4	160.8	164.9
50s	155.9	149.2	153.2	158.6	162.7
